# Targeting subchondral bone mesenchymal stem cell activities for intrinsic joint repair in osteoarthritis

**DOI:** 10.4155/fsoa-2017-0055

**Published:** 2017-09-06

**Authors:** Dragos C Ilas, Sarah M Churchman, Dennis McGonagle, Elena Jones

**Affiliations:** 1 Leeds Institute of Rheumatic & Musculoskeletal Medicine, The University of Leeds, Leeds, UK; 2 NIHR-Leeds Musculoskeletal Biomedical Research Unit, Chapel Allerton, Leeds Teaching Hospitals NHS Trust, Leeds, UK

**Keywords:** bone remodeling, mesenchymal, osteoarthritis, stem cells, subchondral bone

## Abstract

Osteoarthritis (OA) is a common age-related disease with complex pathophysiology. It is characterized by wide-ranging tissue damage and ultimate biomechanical failure of the whole joint. However, signs of tissue adaptation and attempted repair responses are evident in OA-affected osteochondral tissues. Highlighted in this review article is the role of bone-resident mesenchymal stem cells (MSCs) in these bone remodeling responses, and a proposal that targeting MSC activities in OA subchondral bone could represent a novel approach for intrinsic joint regeneration in OA. The development of these therapies will require better understanding of MSC proliferation, migration and differentiation patterns in relation to OA tissue damage and further clarification of the molecular signaling events in these MSCs during disease progression.

## Lay abstract

Osteoarthritis (OA) is a joint disorder, in which the cartilage, the underlying bone and other joint tissues are affected. Recent evidence demonstrating attempted repair responses in these OA tissues challenges the traditional view of OA as a degenerative disorder. Signs of tissue regeneration are particularly evident in the bone located directly underneath the damaged cartilage, where increased stem cell activity has been observed. Targeting these stem cells could represent a novel approach for intrinsic joint regeneration in OA. To progress with developing these novel therapies, a better understanding of stem cell function in normal and OA joint tissues is needed.

## Clinicopathological considerations

Osteoarthritis (OA) is the most common form of chronic joint disease in man and represents the leading cause of pain and disability with increased prevalence due to aging populations [1]. It has a complex pathophysiology and in its final stages it is characterized by chronic pain and stiffness, which is linked to variable levels of inflammation and severe cartilage loss, all ultimately leading to joint failure. Despite the high prevalence of OA and its association with the aging process [2], it still remains an idiopathic and poorly understood condition. OA is an active and progressive disease, which in comparison to rheumatoid arthritis has no effective disease-modifying drug therapy. At the clinical stage, conventional symptomatic treatments (pain analgesics, intra-articular corticosteroids injections, glucosamine and/or chondroitin sulfate, chondroprotective drugs) have little effect on OA progression, with joint replacement ultimately being the only option to restore function and alleviate pain.

OA has long been considered a disease of the cartilage but now it is acknowledged as a disease of the whole joint, including closely linked alterations in cartilage, synovium and subchondral bone [3]. The synovium consists of the synovial membrane which encapsulates the joint providing structural support, synovial fluid for appropriate lubrication and nutrients essential for normal joint function [4]. Subchondral bone has been defined in a number of ways but is generally referred to as any bone component distal to calcified cartilage and is separated into two separate anatomic entities: the subchondral plate, consisting of cortical bone underlying the calcified cartilage and the subchondral trabecular bone [5].

**Figure 1.**
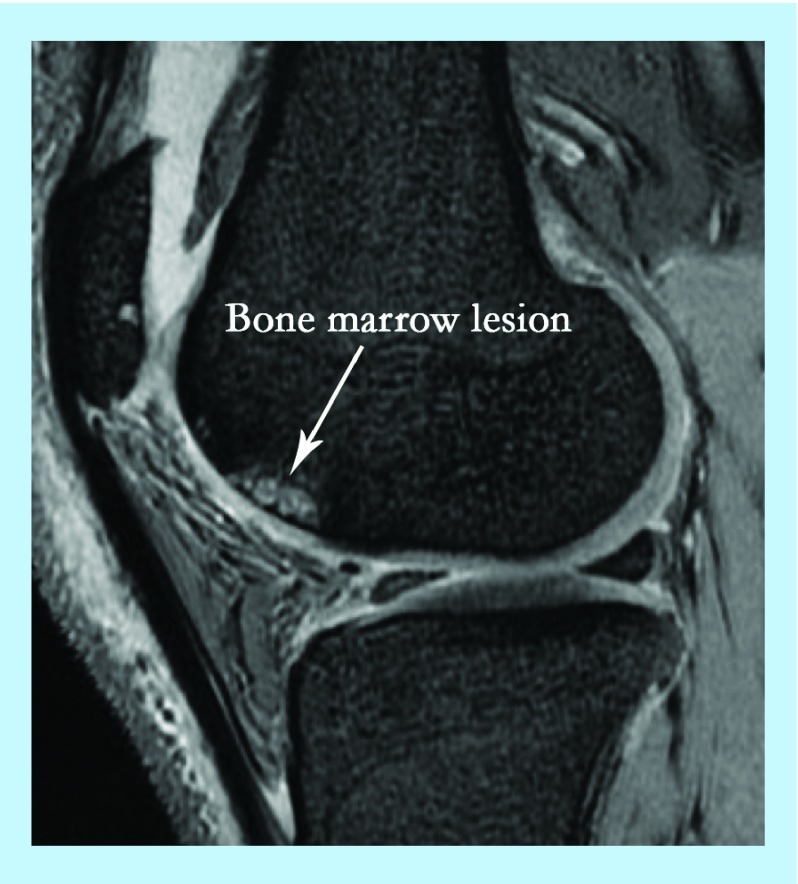
**MRI of knee osteoarthritis showing bone marrow lesion.**

It is now recognized that the OA process can start in various joint structures including ligaments, menisci or in the bone where the latter has been termed as osteogenic OA [6]. Whether cartilage damage in OA affects the underlying bone or vice versa is still a matter of debate but emerging evidence emphasizes the importance of bone involvement in OA progression. For example, some of the hallmarks of OA include an increase in bone volume and the thickness of subchondral bone [7] as well as the increased number of trabeculae and altered mineralization [8,9]. Another important feature implicating bone involvement in OA is the formation of bone cysts, and the appearance of bone marrow (BM) lesions and osteophytes [5]. BM lesions (BMLs) are usually identified by ill-defined high signal on MRI and comprise of edema, necrosis and fibrosis (Figure 1). They have received increased attention lately due to their ‘almost exclusive’ association with cartilage degradation and alteration of the articular surface in OA [10]. Osteophytes on the other hand, are fibrocartilage-capped bony outgrowths and a source of pain and loss of joint function [11]. It remains unclear whether the occurrence of all of these bone changes in OA is due to a pathological process or functional adaptation.

## Mesenchymal stem cells & bone remodeling in OA

**Figure 2.**
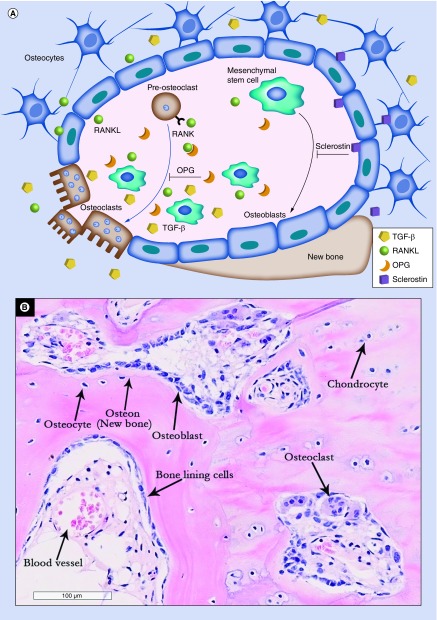
**Bone remodeling process.** **(A)** Signals involved in bone remodeling and the role of mesenchymal stem cells (MSCs) in these processes. MSCs are precursors of osteoblasts (black arrow). The process of osteoblastogenesis is inhibited by sclerostin produced by osteocytes. In concert with osteocytes, MSCs also control osteoclastogenesis via the production of RANKL and OPG (blue arrow). In OA, osteoclasts release TGF-β from the bone matrix, which modulates MSC activity near the resorption site. **(B)** Bone remodeling activity in OA subchondral bone (hematoxylin and eosin staining of EDTA-decalcified femoral head subchondral bone). OA: Osteoarthritis.

During disease progression, the aforementioned structural changes in OA bone are a predictable consequence of alterations in the cell-mediated bone remodeling process. Bone remodeling is an active and dynamic process that depends upon the tightly regulated balance between bone formation and bone resorption [12]. The key cellular players in bone remodeling are osteoclasts; the main bone resorbing cells and osteoblast lineage cells; mesenchymal stem/stromal cells (MSCs) and their descendants, osteoblasts and osteocytes (Figure 2A). The molecular communication between the cells involved in this coordinated process plays a pivotal role in maintaining bone homeostasis and repair [13]. Bone remodeling is believed to commence at the inner bone surface where signals induced by microdamage or increased mechanical load initiate bone resorption by the osteoclasts. Bone resorption is regulated by a balanced signaling pathway composed of OPG, RANKL and its receptor; RANK [14]. Following bone resorption, abundant factors such as TGF-β [15] and IGF-1 [16] are secreted into the BM environment and recruit MSCs to the remodeling site where they progressively commit to the osteoblast lineage for subsequent new bone formation, thus coupling bone remodeling spatially and temporally [17]. Bone remodeling activity in OA subchondral bone is shown on Figure 2B.

MSCs were first described as a rare BM-derived adherent population of colony-forming cells with high proliferative capacity [18]. BM MSCs have multidirectional differentiation potentials, including osteogenic, chondrogenic and adipogenic and are critical to the bone microenvironment by virtue of their roles in bone remodeling and supporting hematopoiesis [19]. During bone remodeling, MSCs are able to produce soluble OPG, which acts as a decoy for RANKL by binding to its receptor; RANK, thus inhibiting osteoclastogenesis [20] suggesting their important role not only in bone formation, but also in bone resorption (Figure 2A). In addition, MSCs are immunomodulatory cells able to regulate adaptive and innate immune responses, which in the BM microenvironment could be seen as their control of immune cell egress into systemic circulation as well as being a trigger for ‘emergency’ myelopoiesis in response to acute infection [21]. MSCs are fairly abundant in human trabecular [22,23] and cortical bone cavities [24], and in the trabecular subchondral bone in OA [25].

**Figure 3.**
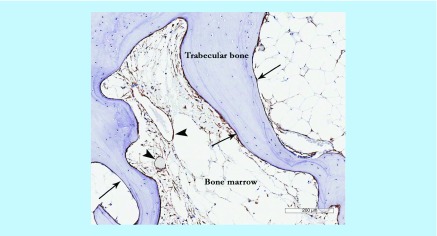
**Mesenchymal stem cell appearance in osteoarthritis subchondral bone showing CD271^+^ staining in perivascular (arrow heads) and bone lining (arrows) locations particularly in the areas of newly-formed bone.**

There are internationally recognized criteria for defining culture-expanded MSCs [26]. However, culture-expanded cells are not functionally and transcriptionally equivalent to their *in vivo* counterparts, notably with regard to bone-related signaling pathways [27], adhesion receptors and signaling molecules [28] and homing capacity [29]. So far the low-affinity nerve growth factor receptor/CD271 is considered one of the most specific and reliable markers for native human BM MSCs [30]. In terms of their topographical distribution in BM cavities, the CD271-positive cells occupy bone lining and perivascular niches, the latter characterized by the co-expression of the CD146 marker, in addition to CD271 [31,32]. In OA, the numbers of CD271-positive cells are increased [25], particularly around the areas of newly formed bone (Figure 3).

MSCs cultured from mouse BM or compact bone share the same surface markers, being positive for CD29, CD44, CD105 and Sca-1 expression [33]. PDGFR-α and Sca-1 in particular, have been suggested as selective markers of mouse BM MSCs [29] and commonly used for their prospective isolation from mouse BM [34]. However, an intracellular intermediate filament, nestin, remains up to now the most commonly used marker of mouse BM MSCs in many animal model studies [35,36].

## MSCs & their progeny in OA bone

MSCs are common residents of all joint tissues, including synovial tissue and fluid [37] as well as bone [23,24]. Since the focus of this review is OA subchondral bone compartment, and MSCs found in other joint tissues have been extensively reviewed elsewhere [38], the following discussion will be specific to MSCs resident in the BM or bone of OA patients.

One of the earliest studies on this issue was carried out by Murphy *et al*. which reported that MSCs in OA BM remote from the site of damage had reduced chondrogenic and adipogenic potentials [39]. A more recent OA animal study, in which anterior cruciate ligament transection was used to induce OA and nestin was employed as a marker for identifying and tracking MSCs, has shown their accumulation and deranged differentiation in OA affected joints and suggested to be a result of the altered TGF-β availability in these areas [40]. The high concentrations of TGF-β are released from the subchondral bone during osteoclast-mediated bone resorption, facilitating MSCs recruitment [41] (Figure 2A). Interestingly, these alterations in TGF-β availability may be also be responsible for osteophyte formation in OA. Indeed, an important role in osteophytes development has been similarly attributed to abnormal mechanical load and TGF-β release [11]. In agreement with the Zhen *et al*. study, Campbell *et al*. have recently shown that the number of resident MSCs in affected human OA bone (in the BML areas) was also increased compared with non-BML areas but their proliferation and mineralization capacities were reduced [25]. Altered gene expression profiles were also observed in MSCs culture expanded from OA BMLs compared with nonlesional areas, including RANKL expression [25]. Further studies are needed to validate these findings using purified uncultured MSCs from OA bone but in accumulation, the available evidence thus far points toward the notion that OA progression impacts MSC physiology in the affected area and that MSC loss of function may in fact play a major role in the failed joint repair responses in OA.

Similarly, there is increasing evidence for the abnormal behavior and phenotypes of osteoblasts in OA joints [42,43]. Based on endogenous production of PGE_2_ by their osteoblasts, OA patients have been categorized into two groups: low PGE_2_ and high PGE_2_ [44,45]. Low PGE_2_ osteoblasts were shown to favor bone resorption having a low OPG/RANKL ratio due to high RANKL levels compared with normal osteoblasts, which also correlated with reduced subchondral bone mass in these patients. In contrast, the high PGE_2_ groups showed an increase in subchondral bone thickness and higher OPG/RANKL ratio in osteoblasts [44]. Osteoarthritic osteoblasts were also reported to have increased levels of TGF-β [46], alkaline phosphatase, osteocalcin and *COL1A1/COL1A2* mRNA ratio [47] as well as altered mineralization potential and a dramatic variation of calcium/phosphate ratio [48].

Further down osteogenesis pathway, the osteocytes; that represent embedded and terminally differentiated osteoblasts, also control bone remodeling including both formation and resorption [49]. They are the main source of RANKL [50] indicating their major role in bone resorption (Figure 2A). Indeed, mouse model studies provided strong evidence that the osteocyte network enhances bone resorption and inhibits bone formation under physiological conditions [51]. RANKL expression is further induced in the viable osteocytes neighboring the site of microdamage [52] leading to increased osteoclast formation at these sites. Another phenomenon, osteocytic osteolysis that refers to osteocytes’ own potential to remodel their perilacunar and canalicular matrix by removing and replacing bone, represents a direct mechanism by which osteocytes can resorb bone and actively contribute to mineral homeostasis [53,54]. In a recent study perilacunar remodeling dysregulation was shown to be important in the pathogenesis of glucocorticoid-induced osteonecrosis [55].

There is not yet enough evidence to support the idea that osteocytic osteolysis is involved in OA pathophysiology, but due to the extent of the osteocytes network, this process in our opinion merits further consideration. On the other hand, osteocyte apoptosis is well recognized in OA as well as abnormal osteocyte morphology [48]. Given the high bone area covered through their lacuno–canalicular network [49] and its vital role for osteocytes’ survival by providing their nutrients and communication, any changes in the osteocyte network integrity may play a considerable role in OA progression.

Taking this evidence together, it is possible to suggest that any alterations in MSCs in OA can affect their descendants in bone chiefly osteoblasts, which are short-living cells immediately involved in bone remodeling. Osteocytes are longer-living cells [56] and although they are descendants of MSCs, changes in their behavior during OA progression could be less attributable to the abnormal behavior of MSCs, and be more related to altered biomechanical loads. Nevertheless, because MSCs are also the progenitors for other lineages (adipogenic and chondrogenic in addition to osteogenic), this further emphasizes that MSCs can be regarded as a critical cellular player in the bone microenvironment in OA.

## Therapies aimed to restore MSC bone remodeling & other activities in OA

As mentioned above, subchondral bone abnormalities observed in OA, such as subchondral plate thickening, osteophyte formation and particularly BMLs, which are characterized by thicker but less mineralized softer bone, suggest that the OA process is associated with altered bone remodeling activity around the site of damage. These changes are often accompanied by raised concentrations of bone degradation products in the blood of OA patients, such as N- and C-terminal type I collagen telopeptides or amino- and carboxyl-terminal procollagen propeptides of type I collagen; however the value of measuring these molecules alongside cartilage degradation products as OA disease biomarkers remains debatable [3]. As the frequency of BMLs increases with disease progression [57], and subchondral bone MSCs appear to accumulate in these areas, therapeutic interventions aiming to correct MSC behavior in OA bone could theoretically prevent OA progression. In the sections below we describe how our current understanding of the state of MSCs and their descendants in OA subchondral bone could lead to novel approaches for OA treatment.

### Common & new-generation antiresorptives

Common antiresorptive agents such as bisphosphonates have been used in many animal models of OA. While these studies have demonstrated several improvements in disease activity [58], the clinical results are less clear [59–61]. In contrast to osteoporosis in which osteoclast-driven bone resorption is the pathological process being targeted, OA is characterized by ‘the spatial and temporal separation of these processes during joint degeneration’ (increased remodeling during early disease and its reduced rate as the disease progresses) [9]. A more rationalized use of bisphosphonates in OA therefore requires better understanding of the ‘window of opportunity’ in which these antiresorptive agents could lead to restoration of normal bone homeostasis, and should take into consideration their effects on MSCs [62].

Viewing MSCs as central players in controlling bone formation and remodeling as well as hemopoiesis at the site of OA, it is conceivable that dual action agents such as strontium ranelate may more effectively interfere with the development of BMLs [63], by reducing excessive local osteoclast activity and also promoting mineralization of the newly formed bone. Indeed, two recent clinical trials have shown positive structural effects of strontium ranelate on knee OA, one improving the joint space narrowing [64] and the other reducing the loss of cartilage volumes concurrent with the decrease of BMLs at 3 years of follow-up [65]. Other antiresorptive agents such as cathepsin K inhibitors, that target the resorption process specifically without affecting the osteoclasts [66] have shown promising results in OA animal models [67,68]. These are already in Phase II and III clinical trials for osteoporosis [69] and could therefore be considered for clinical trials in patients with early OA. Further work is needed to better understand the effects of all of these agents on the signaling pathways occurring in OA subchondral bone MSCs.

### Targeting TGF-β & sclerostin

As previously mentioned, several groups have identified TGF-β as a target molecule for OA modulation [40,70]. It has been shown to be overproduced by OA osteoblasts and is actively involved in the induction and development of osteophytes [71]. Based on their previous ground-breaking study [40] on the effects of TGF-β on subchondral bone MSCs in OA, Zhen *et al*. subsequently demonstrated that the systemic use of a TGF-β-neutralizing antibody attenuated OA progression in rodent OA models [72]. However, there remains a concern that such TGF-β inhibition may simultaneously exacerbate cartilage degeneration in OA. In normal joints, TGF-β levels are tightly controlled in order to maintain healthy cartilage and simultaneously avoid joint fibrosis, osteophyte formation and other pathological subchondral bone changes. Therefore, further development of anti-TGF-β therapeutics in OA would require a careful dose titration to ensure no damage to the cartilage and ideally, attenuation of its degeneration [72]. This emphasizes the importance of targeting the osteochondral unit as a whole in OA, as well as both subchondral bone and overlaying cartilage components as separate entities [9,73]. This is particularly pertinent for advanced OA, in which the osteochondral junction is breached thus allowing enhanced molecular and cellular communication between these two tissues.

TGF-β is known to cooperate with Wnt signaling to stimulate both osteoblast and chondrocyte differentiation in a positive regulatory loop [74]. A major inhibitor of Wnt signaling and consequently, bone formation, is sclerostin (Figure 2A). Exclusively secreted by osteocytes and hypertrophic chondrocytes, its role in OA is still under debate. Sclerostin inhibition is currently being evaluated for the treatment of postmenopausal osteoporosis in humans [75] but its use in OA is controversial. Although it was shown that its loss did not affect cartilage remodeling in a mouse model of OA [76], other studies have shown that it can promote OA [77]. A recent study by Wehmeyer *et al*. raised more concerns when the inhibition of sclerostin led to increased inflammation and joint destruction in a TNF-dependent rheumatoid arthritis mouse model [78]. Further work is needed to establish the main cellular targets for sclerostin and its specific effects on subchondral bone MSCs.

### Targeting MSC migration

A number of interesting observations describing migration of cells, including MSCs, across the damaged subchondral junction and into the synovial fluid [79,80], has led to the development of OA therapies targeting MSC migration. These studies alluded to the fact that MSC migration from the subchondral bone toward the joint surface may represent an attempted repair response, and be a factor contributing to increased abundance of MSCs in OA synovial fluid [80]. Of note, MSC migration from the subchondral bone into a natural fibrin clot is believed to be the main mechanism for fibrocartilaginous cartilage repair following microfracture [81], a common procedure for isolated cartilage defect treatment in early OA.

More recent evidence suggests that migration of MSCs along chemokine gradients created along thinned cartilage fissures and other breaks in the osteochondral junction, may acquire undesirable features such as the loss of chondrogenic potential [80], thus explaining why these attempts to repair are inefficient. Another study recently demonstrated chemokine receptor alterations in MSCs derived from BML areas of OA bone, including *CXCR4* (the receptor for SDF-1α) [25], suggesting their increased sensitivity to local chemokine gradients emanating from the synovial fluid. Because SDF-1α levels are increased in OA synovial fluid [82] and it is a catabolic factor for cartilage homeostasis, inhibition of SDF-1α/CXCR4 signaling has been recently attempted in post-traumatic OA mouse model showing a partial prevention of bone loss and cartilage degeneration [83]. Future clinical studies are needed to validate this concept in humans.

Aberrant migration and escape of endogenous subchondral bone MSCs into the synovial fluid can be also achieved with the use of barrier membranes in procedures such as matrix-assisted chondrocyte implantation [84], which may be another mechanism contributing to neocartilage tissue formation in addition to exogenous chondrocytes.

### Platelet-rich plasma based approaches

Intra-articular injections of platelet-rich plasma (PRP) have been used in sports medicine for a great number of years and have shown mixed results, primarily due to a lack of consistency in PRP preparation, characterization and administration [85,86]. An interesting recent development has been the pioneering administration of intra-osseous PRP for the treatment of cartilage defects in OA [87]. The mechanism of PRP action in these reports remains unclear but it is possible to suggest that its direct administration into OA bone enables better retention and higher *in situ* concentrations of PRP constituents in subchondral bone areas, allowing more direct action on subchondral bone MSCs. Active PRP components that may exert beneficial effects on MSCs could include platelet-derived growth factor, known to enhance MSC proliferation *in vivo* [88,89], as well as chemokines and anti-inflammatory molecules such as interleukin receptor antagonist [90], which can influence MSCs retention and their immunomodulatory activity in subchondral bone areas. More work is needed to dissect the mechanisms of action of these different molecules on MSCs in the OA bone toward better understanding and a more controlled use of these biological autologous therapies for OA management.

### Targeting BMLs

As MSC abnormalities appear to be most confined to the BML areas of OA bone, these BMLs may be targeted for direct administration of therapeutic agents into OA bone. While it may be seen as rather invasive in the OA rheumatology community, MSC and scaffold delivery to the lesional areas of bone is a common procedure for the treatment of avascular necrosis of the femoral head in the orthopedic field [91]. Furthermore, a novel procedure termed ‘subchondroplasty’, the delivering of bone fillers and bone matrix substitutes into the BML areas of bone in knee OA is being increasingly used [92]. While these bone substitutes are aimed at preventing bone collapse following BML development, it is conceivable that bone matrix loaded with PRP or above-mentioned pharmacological compounds, may lead to improvement of bone homeostasis in BMLs and therefore, to longer joint preservation.

It is known that BML volumes change over the course of repeated MRI investigations [93]. This could be due to the already mentioned intermittent changes in the remodeling activity of subchondral bone MSCs as well as their previously unappreciated high migratory ability [40]. BML-targeted therapies may therefore be most effective if combined with intra-articular PRP injections, as shown in a recent study [94], or as an adjunct therapy to current cartilage resurfacing interventions.

## Conclusion

Current therapies for OA treatment focus either on the addition of exogenous MSCs to the affected joint or the use of scaffolds, with or without MSCs, in order to plug cartilage defects and restore joint surface continuity [84]. While recognizing the importance of subchondral bone abnormalities in OA development, current understanding of the pathophysiology of subchondral bone MSCs in OA and appreciation of their potential as target cells for future OA therapies remains limited. Future efforts need to be directed toward better understanding of how bone-resident MSCs respond to early and later drivers of OA such as biomechanical and biochemical changes in the bone's extracellular matrix or the effects of synovial fluid constituents including pro-inflammatory cytokines and chemokines, on these MSCs. Having identified potential MSC modifiers such as TGF-β and SDF-1α, next steps would require development of optimal routes and procedures for their administration that would be as a consequence of a better appreciation of OA as a disease of the whole joint.

These therapies are likely to act on a broader range of cells, not only MSCs, therefore future efforts should present a holistic approach to subchondral bone OA treatment whereby both mesenchymal- and hematopoietic-lineage stem cells and their descendants are all considered for simultaneous modulation, taking into account their native niches and three-dimensional environments.

## Future perspective

In contrast to osteoporosis, which is a systemic bone disease, OA is characterized by local and temporal alterations in bone remodeling activity, in which MSCs appear to have a prominent role. Future joint regeneration strategies should not only aim at restoration of the damaged cartilage, but also at target subchondral bone MSC abnormalities, by means of pharmacological and biological agents to correct their altered migration, proliferation and differentiation capacities. These interventions may be most successful in conjunction with novel surgical techniques such as subchondroplasty.

**Executive summary**

- There are spatial and temporal alterations in the bone remodeling processes of osteoarthritis (OA) subchondral bone that can be therapeutically targeted, in order to restore osteochondral tissue homeostasis.
- Recent evidence demonstrates significant changes in mesenchymal stem cell migration, proliferation and differentiation potentials in OA subchondral bone, particularly in its damaged areas.
- Correcting mesenchymal stem cell functionality in disease-affected areas of OA bone can represent a novel strategy for joint regeneration in OA.

## References

[1] Golightly YM, Allen KD, Jordan JM (2016). Defining the burden of osteoarthritis in population-based surveys. *Arthritis Care Res. (Hoboken)*.

[2] Loeser RF (2013). Aging processes and the development of osteoarthritis. *Curr. Opin. Rheumatol.*.

[3] Sharma AR, Jagga S, Lee SS, Nam JS (2013). Interplay between cartilage and subchondral bone contributing to pathogenesis of osteoarthritis. *Int. J. Mol. Sci.*.

[4] Smith MD (2011). The normal synovium. *Open Rheumatol. J.*.

[5] Li G, Yin J, Gao J (2013). Subchondral bone in osteoarthritis: insight into risk factors and microstructural changes. *Arthritis Res. Ther.*.

[6] Mcgonagle D, Tan AL, Carey J, Benjamin M (2010). The anatomical basis for a novel classification of osteoarthritis and allied disorders. *J. Anat.*.

[7] Goldring MB, Goldring SR (2010). Articular cartilage and subchondral bone in the pathogenesis of osteoarthritis. *Ann. NY Acad. Sci.*.

[8] Bettica P, Cline G, Hart DJ, Meyer J, Spector TD (2002). Evidence for increased bone resorption in patients with progressive knee osteoarthritis: longitudinal results from the Chingford study. *Arthritis Rheum.*.

[9] Burr DB, Gallant MA (2012). Bone remodelling in osteoarthritis. *Nat. Rev. Rheumatol.*.

[10] Bowes MA, Mclure SW, Wolstenholme CB (2016). Osteoarthritic bone marrow lesions almost exclusively colocate with denuded cartilage: a 3D study using data from the Osteoarthritis initiative. *Ann. Rheum. Dis.*.

[11] Wong SH, Chiu KY, Yan CH (2016). Review article: osteophytes. *J. Orthop. Surg. (Hong Kong)*.

[12] Rucci N (2008). Molecular biology of bone remodelling. *Clin. Cases Miner. Bone Metab.*.

[13] Nakahama K (2010). Cellular communications in bone homeostasis and repair. *Cell Mol. Life Sci.*.

[14] Charles JF, Aliprantis AO (2014). Osteoclasts: more than ‘bone eaters’. *Trends Mol. Med.*.

[15] Crane JL, Cao X (2014). Bone marrow mesenchymal stem cells and TGF-beta signaling in bone remodeling. *J. Clin. Invest.*.

[16] Xian L, Wu X, Pang L (2012). Matrix IGF-1 maintains bone mass by activation of mTOR in mesenchymal stem cells. *Nat. Med.*.

[17] Crane JL, Cao X (2014). Function of matrix IGF-1 in coupling bone resorption and formation. *J. Mol. Med. (Berl.)*.

[18] Bianco P, Robey PG (2015). Skeletal stem cells. *Development*.

[19] Greenbaum A, Hsu YM, Day RB (2013). CXCL12 in early mesenchymal progenitors is required for haematopoietic stem-cell maintenance. *Nature*.

[20] Oshita K, Yamaoka K, Udagawa N (2011). Human mesenchymal stem cells inhibit osteoclastogenesis through osteoprotegerin production. *Arthritis Rheum.*.

[21] Ziegler P, Boettcher S, Takizawa H, Manz MG, Brummendorf TH (2016). LPS-stimulated human bone marrow stroma cells support myeloid cell development and progenitor cell maintenance. *Ann. Hematol.*.

[22] Sakaguchi Y, Sekiya I, Yagishita K, Ichinose S, Shinomiya K, Muneta T (2004). Suspended cells from trabecular bone by collagenase digestion become virtually identical to mesenchymal stem cells obtained from marrow aspirates. *Blood*.

[23] Jones E, English A, Churchman SM (2010). Large-scale extraction and characterization of CD271^+^ multipotential stromal cells from trabecular bone in health and osteoarthritis: implications for bone regeneration strategies based on uncultured or minimally cultured multipotential stromal cells. *Arthritis Rheum.*.

[24] Cox G, Boxall SA, Giannoudis PV (2012). High abundance of CD271(+) multipotential stromal cells (MSCs) in intramedullary cavities of long bones. *Bone*.

[25] Campbell TM, Churchman SM, Gomez A (2016). Mesenchymal stem cell alterations in bone marrow lesions in hip osteoarthritis. *Arthritis Rheumatol. (Hoboken, NJ)*.

[26] Dominici M, Le Blanc K, Mueller I (2006). Minimal criteria for defining multipotent mesenchymal stromal cells. The International Society for Cellular Therapy position statement. *Cytotherapy*.

[27] Churchman SM, Ponchel F, Boxall SA (2012). Transcriptional profile of native CD271^+^ multipotential stromal cells: evidence for multiple fates, with prominent osteogenic and Wnt pathway signaling activity. *Arthritis Rheum.*.

[28] Qian H, Le Blanc K, Sigvardsson M (2012). Primary mesenchymal stem and progenitor cells from bone marrow lack expression of CD44 protein. *J. Biol. Chem.*.

[29] Morikawa S, Mabuchi Y, Kubota Y (2009). Prospective identification, isolation, and systemic transplantation of multipotent mesenchymal stem cells in murine bone marrow. *J. Exp. Med.*.

[30] Boxall SA, Jones E (2012). Markers for characterization of bone marrow multipotential stromal cells. *Stem Cells Int.*.

[31] Tormin A, Li O, Brune JC (2011). CD146 expression on primary nonhematopoietic bone marrow stem cells is correlated with *in situ* localization. *Blood*.

[32] Sacchetti B, Funari A, Michienzi S (2007). Self-renewing osteoprogenitors in bone marrow sinusoids can organize a hematopoietic microenvironment. *Cell*.

[33] Li H, Ghazanfari R, Zacharaki D, Lim HC, Scheding S (2016). Isolation and characterization of primary bone marrow mesenchymal stromal cells. *Ann. NY Acad. Sci.*.

[34] Houlihan DD, Mabuchi Y, Morikawa S (2012). Isolation of mouse mesenchymal stem cells on the basis of expression of Sca-1 and PDGFR-alpha. *Nat. Protoc.*.

[35] Mendez-Ferrer S, Michurina TV, Ferraro F (2010). Mesenchymal and haematopoietic stem cells form a unique bone marrow niche. *Nature*.

[36] Pinho S, Lacombe J, Hanoun M (2013). PDGFRalpha and CD51 mark human nestin+ sphere-forming mesenchymal stem cells capable of hematopoietic progenitor cell expansion. *J. Exp. Med.*.

[37] Roberts S, Genever P, Mccaskie A, De Bari C (2011). Prospects of stem cell therapy in osteoarthritis. *Regen. Med.*.

[38] Barry F, Murphy M (2013). Mesenchymal stem cells in joint disease and repair. *Nat Rev Rheumatol.*.

[39] Murphy JM, Dixon K, Beck S, Fabian D, Feldman A, Barry F (2002). Reduced chondrogenic and adipogenic activity of mesenchymal stem cells from patients with advanced osteoarthritis. *Arthritis Rheum.*.

[40] Zhen G, Wen C, Jia X (2013). Inhibition of TGF-β signaling in mesenchymal stem cells of subchondral bone attenuates osteoarthritis. *Nat. Med.*.

[41] Tang Y, Wu X, Lei W (2009). TGF-beta1-induced migration of bone mesenchymal stem cells couples bone resorption with formation. *Nat. Med.*.

[42] Goldring MB (2012). Chondrogenesis, chondrocyte differentiation, and articular cartilage metabolism in health and osteoarthritis. *Ther. Adv. Musculoskelet. Dis.*.

[43] Sanchez C, Deberg MA, Bellahcene A (2008). Phenotypic characterization of osteoblasts from the sclerotic zones of osteoarthritic subchondral bone. *Arthritis Rheum.*.

[44] Tat SK, Pelletier JP, Velasco CR, Padrines M, Martel-Pelletier J (2009). New perspective in osteoarthritis: the OPG and RANKL system as a potential therapeutic target?. *Keio J. Med.*.

[45] Kwan Tat S, Pelletier JP, Lajeunesse D, Fahmi H, Lavigne M, Martel-Pelletier J (2008). The differential expression of osteoprotegerin (OPG) and receptor activator of nuclear factor kappaB ligand (RANKL) in human osteoarthritic subchondral bone osteoblasts is an indicator of the metabolic state of these disease cells. *Clin. Exp. Rheumatol.*.

[46] Massicotte F, Lajeunesse D, Benderdour M (2002). Can altered production of interleukin-1beta, interleukin-6, transforming growth factor-beta and prostaglandin E(2) by isolated human subchondral osteoblasts identify two subgroups of osteoarthritic patients. *Osteoarthritis Cartilage*.

[47] Couchourel D, Aubry I, Delalandre A (2009). Altered mineralization of human osteoarthritic osteoblasts is attributable to abnormal Type I collagen production. *Arthritis Rheum.*.

[48] Kumarasinghe DD, Sullivan T, Kuliwaba JS, Fazzalari NL, Atkins GJ (2012). Evidence for the dysregulated expression of TWIST1, TGFbeta1 and SMAD3 in differentiating osteoblasts from primary hip osteoarthritis patients. *Osteoarthritis Cartilage*.

[49] Prideaux M, Findlay DM, Atkins GJ (2016). Osteocytes: the master cells in bone remodelling. *Curr. Opin. Pharmacol.*.

[50] Xiong J, Onal M, Jilka RL, Weinstein RS, Manolagas SC, O'brien CA (2011). Matrix-embedded cells control osteoclast formation. *Nat. Med.*.

[51] Komori T (2013). Functions of the osteocyte network in the regulation of bone mass. *Cell Tissue Res.*.

[52] Kennedy OD, Herman BC, Laudier DM, Majeska RJ, Sun HB, Schaffler MB (2012). Activation of resorption in fatigue-loaded bone involves both apoptosis and active pro-osteoclastogenic signaling by distinct osteocyte populations. *Bone*.

[53] Teti A, Zallone A (2009). Do osteocytes contribute to bone mineral homeostasis? Osteocytic osteolysis revisited. *Bone*.

[54] Wysolmerski JJ (2012). Osteocytic osteolysis: time for a second look?. *Bonekey Rep.*.

[55] Fowler TW, Acevedo C, Mazur CM (2017). Glucocorticoid suppression of osteocyte perilacunar remodeling is associated with subchondral bone degeneration in osteonecrosis. *Sci. Rep.*.

[56] Manolagas SC, Parfitt AM (2010). What old means to bone. *Trends Endocrinol. Metab.*.

[57] Tanamas SK, Wluka AE, Pelletier JP (2010). Bone marrow lesions in people with knee osteoarthritis predict progression of disease and joint replacement: a longitudinal study. *Rheumatology (Oxford)*.

[58] Zhu S, Chen K, Lan Y, Zhang N, Jiang R, Hu J (2013). Alendronate protects against articular cartilage erosion by inhibiting subchondral bone loss in ovariectomized rats. *Bone*.

[59] Davis AJ, Smith TO, Hing CB, Sofat N (2013). Are bisphosphonates effective in the treatment of osteoarthritis pain? A meta-analysis and systematic review. *PLoS ONE*.

[60] Karsdal MA, Bay-Jensen AC, Lories RJ (2014). The coupling of bone and cartilage turnover in osteoarthritis: opportunities for bone antiresorptives and anabolics as potential treatments?. *Ann. Rheum. Dis.*.

[61] Laslett LL, Dore DA, Quinn SJ (2012). Zoledronic acid reduces knee pain and bone marrow lesions over 1 year: a randomised controlled trial. *Ann. Rheum. Dis.*.

[62] Misra J, Mohanty ST, Madan S (2016). Zoledronate attenuates accumulation of DNA damage in mesenchymal stem cells and protects their function. *Stem Cells*.

[63] Han W, Fan S, Bai X, Ding C (2017). Strontium ranelate, a promising disease modifying osteoarthritis drug. *Expert Opin. Investig. Drugs*.

[64] Reginster JY, Badurski J, Bellamy N (2013). Efficacy and safety of strontium ranelate in the treatment of knee osteoarthritis: results of a double-blind, randomised placebo-controlled trial. *Ann. Rheum. Dis.*.

[65] Pelletier JP, Roubille C, Raynauld JP (2015). Disease-modifying effect of strontium ranelate in a subset of patients from the Phase III knee osteoarthritis study SEKOIA using quantitative MRI: reduction in bone marrow lesions protects against cartilage loss. *Ann. Rheum. Dis.*.

[66] Boonen S, Rosenberg E, Claessens F, Vanderschueren D, Papapoulos S (2012). Inhibition of cathepsin K for treatment of osteoporosis. *Curr. Osteoporos. Rep.*.

[67] Mcdougall JJ, Schuelert N, Bowyer J (2010). Cathepsin K inhibition reduces CTXII levels and joint pain in the guinea pig model of spontaneous osteoarthritis. *Osteoarthritis Cartilage*.

[68] Hayami T, Zhuo Y, Wesolowski GA, Pickarski M, Duong LT (2012). Inhibition of cathepsin K reduces cartilage degeneration in the anterior cruciate ligament transection rabbit and murine models of osteoarthritis. *Bone*.

[69] Duong Le T, Leung AT, Langdahl B (2016). Cathepsin K inhibition: a new mechanism for the treatment of osteoporosis. *Calcif. Tissue Int.*.

[70] Blaney Davidson EN, Vitters EL, Van Der Kraan PM, Van Den Berg WB (2006). Expression of transforming growth factor-beta (TGFbeta) and the TGFbeta signalling molecule SMAD-2P in spontaneous and instability-induced osteoarthritis: role in cartilage degradation, chondrogenesis and osteophyte formation. *Ann. Rheum. Dis.*.

[71] Shen J, Li S, Chen D (2014). TGF-beta signaling and the development of osteoarthritis. *Bone Res.*.

[72] Xie L, Tintani F, Wang X (2016). Systemic neutralization of TGF-beta attenuates osteoarthritis. *Ann. NY Acad. Sci.*.

[73] Goldring SR, Goldring MB (2016). Changes in the osteochondral unit during osteoarthritis: structure, function and cartilage-bone crosstalk. *Nat. Rev. Rheumatol.*.

[74] Wu M, Chen G, Li YP (2016). TGF-beta and BMP signaling in osteoblast, skeletal development, and bone formation, homeostasis and disease. *Bone Res.*.

[75] Macnabb C, Patton D, Hayes JS (2016). Sclerostin antibody therapy for the treatment of osteoporosis: clinical prospects and challenges. *J. Osteoporos.*.

[76] Roudier M, Li X, Niu QT (2013). Sclerostin is expressed in articular cartilage but loss or inhibition does not affect cartilage remodeling during aging or following mechanical injury. *Arthritis Rheum.*.

[77] Bouaziz W, Funck-Brentano T, Lin H (2015). Loss of sclerostin promotes osteoarthritis in mice via beta-catenin-dependent and -independent Wnt pathways. *Arthritis Res. Ther.*.

[78] Wehmeyer C, Frank S, Beckmann D (2016). Sclerostin inhibition promotes TNF-dependent inflammatory joint destruction. *Sci. Transl. Med.*.

[79] Koelling S, Kruegel J, Irmer M (2009). Migratory chondrogenic progenitor cells from repair tissue during the later stages of human osteoarthritis. *Cell Stem Cell*.

[80] Harris Q, Seto J, O'brien K (2013). Monocyte chemotactic protein-1 inhibits chondrogenesis of synovial mesenchymal progenitor cells: an *in vitro* study. *Stem Cells*.

[81] Min BH, Choi WH, Lee YS (2013). Effect of different bone marrow stimulation techniques (BSTs) on MSCs mobilization. *J. Orthop. Res.*.

[82] Kanbe K, Takagishi K, Chen Q (2002). Stimulation of matrix metalloprotease 3 release from human chondrocytes by the interaction of stromal cell-derived factor 1 and CXC chemokine receptor 4. *Arthritis Rheum.*.

[83] Dong Y, Liu H, Zhang X (2016). Inhibition of SDF-1alpha/CXCR4 signalling in subchondral bone attenuates post-traumatic osteoarthritis. *Int. J. Mol. Sci.*.

[84] Smith BD, Grande DA (2015). The current state of scaffolds for musculoskeletal regenerative applications. *Nat. Rev. Rheumatol.*.

[85] Dhillon RS, Schwarz EM, Maloney MD (2012). Platelet-rich plasma therapy - future or trend?. *Arthritis Res. Ther.*.

[86] Moraes VY, Lenza M, Tamaoki MJ, Faloppa F, Belloti JC (2014). Platelet-rich therapies for musculoskeletal soft tissue injuries. *Cochrane Database Syst. Rev.*.

[87] Sanchez M, Anitua E, Delgado D (2016). A new strategy to tackle severe knee osteoarthritis: combination of intra-articular and intraosseous injections of platelet rich plasma. *Expert Opin. Biol. Ther.*.

[88] Philippart P, Meuleman N, Stamatopoulos B (2014). *In vivo* production of mesenchymal stromal cells after injection of autologous platelet-rich plasma activated by recombinant human soluble tissue factor in the bone marrow of healthy volunteers. *Tissue Eng. Part A*.

[89] Tan HB, Giannoudis PV, Boxall SA, Mcgonagle D, Jones E (2015). The systemic influence of platelet-derived growth factors on bone marrow mesenchymal stem cells in fracture patients. *BMC Med.*.

[90] Cassano JM, Kennedy JG, Ross KA, Fraser EJ, Goodale MB, Fortier LA (2016). Bone marrow concentrate and platelet-rich plasma differ in cell distribution and interleukin 1 receptor antagonist protein concentration. *Knee Surg. Sports Traumatol. Arthrosc.*.

[91] Banerjee S, Issa K, Pivec R, Kapadia BH, Khanuja HS, Mont MA (2013). Osteonecrosis of the hip: treatment options and outcomes. *Orthop. Clin. North. Am.*.

[92] Cohen SB, Sharkey PF (2016). Subchondroplasty for treating bone marrow lesions. *J. Knee Surg.*.

[93] Felson DT, Parkes MJ, Marjanovic EJ (2012). Bone marrow lesions in knee osteoarthritis change in 6–12 weeks. *Osteoarthritis Cartilage*.

[94] Muinos-Lopez E, Delgado D, Sanchez P (2016). Modulation of synovial fluid-derived mesenchymal stem cells by intra-articular and intraosseous platelet rich plasma administration. *Stem Cells Int.*.

